# Room Temperature Deposition of Nanocrystalline SiC Thin Films by DCMS/HiPIMS Co-Sputtering Technique

**DOI:** 10.3390/nano12030512

**Published:** 2022-02-01

**Authors:** Vasile Tiron, Elena-Laura Ursu, Daniel Cristea, Georgiana Bulai, George Stoian, Teodora Matei, Ioana-Laura Velicu

**Affiliations:** 1 Department of Exact and Natural Sciences, Research Center on Advanced Materials and Technologies (RAMTECH), Institute of Interdisciplinary Research, Alexandru Ioan Cuza University of Iasi, 700506 Iasi, Romania; vasile.tiron@uaic.ro; 2 Centre of Advanced Research in Bionanoconjugates and Biopolymers, Petru Poni Institute of Macromolecular Chemistry, 700487 Iasi, Romania; ursu.laura@icmpp.ro; 3 Department of Materials Science, Faculty of Materials Science and Engineering, Transilvania University, 500068 Brasov, Romania; 4 Science Department, Integrated Center of Environmental Science Studies in the North-Eastern Development Region (CERNESIM), Institute of Interdisciplinary Research, Alexandru Ioan Cuza University of Iasi, 700506 Iasi, Romania; georgiana.bulai@uaic.ro; 5 National Institute of Research and Development for Technical Physics, 700050 Iasi, Romania; gstoian@phys-iasi.ro; 6 Faculty of Physics, Alexandru Ioan Cuza University of Iasi, 700506 Iasi, Romania; dorateom@yahoo.com

**Keywords:** DCMS/HiPIMS co-sputtering, nanocrystalline silicon carbide, coating, hardness, adhesion

## Abstract

Due to an attractive combination of chemical and physical properties, silicon carbide (SiC) thin films are excellent candidates for coatings to be used in harsh environment applications or as protective coatings in heat exchanger applications. This work reports the deposition of near-stoichiometric and nanocrystalline SiC thin films, at room temperature, on silicon (100) substrates using a DCMS/HiPIMS co-sputtering technique (DCMS—direct current magnetron sputtering; HiPIMS—high-power impulse magnetron sputtering). Their structural and mechanical properties were analyzed as a function of the process gas pressure. The correlation between the films’ microstructure and their mechanical properties was thoroughly investigated. The microstructure and morphology of these films were examined by appropriate microscopic and spectroscopic methods: atomic force microscopy (AFM), scanning electron microscopy (SEM), energy-dispersive X-ray spectroscopy (EDX), X-ray diffraction (XRD), and Raman spectroscopy, while their mechanical and tribological properties were evaluated by instrumented indentation and micro-scratch techniques. The lowest value of the working gas pressure resulted in SiC films of high crystallinity, as well as in an improvement in their mechanical performances. Both hardness (H) and Young’s modulus (E) values were observed to be significantly influenced by the sputtering gas pressure. Decreasing the gas pressure from 2.0 to 0.5 Pa led to an increase in H and E values from 8.2 to 20.7 GPa and from 106.3 to 240.0 GPa, respectively. Both the H/E ratio and critical adhesion load values follow the same trend and increase from 0.077 to 0.086 and from 1.55 to 3.85 N, respectively.

## 1. Introduction

Silicon carbide (SiC) is a semiconductor composed of light elements. Due to its attractive properties, including its low coefficient of thermal expansion, low neutron absorption cross-section, high thermal conductivity, high hardness, and superior tribological, chemical and oxidation resistance, it is well suited as a protective coating in heat exchanger applications [1] or as a sensing material in harsh environments [2,3]. Due to their chemical inertness, SiC-based thin films have great potential as protective layers for Si-based micro-electro-mechanical systems (MEMS) [4,5], coatings for biomedical applications [6], passivation coatings on metallic materials [7], or barrier layers for solar cells [8].

Silicon carbide is also characterized by a relatively wide bandgap, high electron saturated drift velocity, high breakdown field, and good thermal conductivity [9], and it is generally used for high-frequency, high-current-power, and high-temperature electronic devices. To obtain chemically resistant diffusion barriers or passivation layers, pinhole-free films characterized by good uniformity and low stress are required. Compared to the most often used deposition techniques (i.e., pulsed laser deposition and chemical vapor deposition), magnetron sputtering has several important advantages, such as its cost-effectiveness, simplicity, high deposition rate, low deposition temperature, good adhesion of films to the substrate, excellent uniformity on large-area substrates and the ability to easily control films’ stoichiometry [10,11].

Due to a high ionization degree of the sputtered material, the high-power impulse magnetron sputtering (HiPIMS) deposition method has been reported to form smooth, dense, and strongly-adherent materials in the form of thin films/coatings [12,13,14]. Due to its low duty cycle, HiPIMS can operate under a low average power without compromising the ion-to-neutral flux fraction [15]. This feature recommends using HiPIMS in co-sputtering systems when the stoichiometry of the deposited coatings needs to be tuned with high precision or when dealing with a significant difference between the sputtering yields of the targets. In addition, due to its ability to generate energetic gas and metal ion flux [16], HiPIMS is an efficient tool in co-sputtering systems [17], even when the second magnetron is powered in DC mode [18]. As compared to the DCMS technique, due to a higher ion-to-neutral flux ratio and higher kinetic energy of sputtered particles, the coatings deposited by HiPIMS present higher density and demonstrate better adhesion on the substrate. Recently, Galvão et al. have shown that HiPIMS can be used to fabricate nanocrystalline SiC thin films without heating or biasing the Si substrate by making deposits on top of AlN interlayers [19].

To enhance the long-term performance of surface-engineered systems, the mechanical and tribological parameters (i.e., elastic modulus, hardness, and scratch/wear resistance) need to be optimized by varying the process parameters. Despite the enormous potential of silicon carbide films deposited by physical vapor deposition (PVD) systems, as far as we are aware, there is a general lack of relevant information needed to understand the influence of the working gas pressure on their structural and mechanical properties. For this reason, the influence of the deposition process (i.e., working gas pressure) on the properties of SiC films must be studied.

Due to the preferential re-sputtering phenomenon of Si atoms from SiC films, the stoichiometry cannot be fully transported from the target onto the thin film when a SiC target is sputtered [20]. Consequently, for this work, in order to control the chemical composition of the coatings, silicon carbide films were prepared by the DCMS/HiPIMS co-sputtering technique at room temperature by sputtering graphite and silicon targets in direct current magnetron sputtering (DCMS) and HiPIMS modes, respectively. Although the chemical composition of the SiC films can be finely tuned by proper adjustment of the average discharge power applied on each target or through the relative position of the magnetrons, this work reports only results on near-stoichiometric SiC (Si/C atomic ratio of about 1:1).

Hereinafter, the influence of the working gas pressure on the structure, surface morphology, Young’s modulus, hardness, critical adhesion loads, and friction coefficient of SiC thin films obtained by the DCMS/HiPIMS co-sputtering technique will be presented.

## 2. Materials and Methods

### 2.1. Sample Preparation

SiC thin films (thickness of about 750 nm) were deposited on Si (n-type 100) wafers, with a surface area of 20 × 20 mm^2^ and thickness of 0.675 mm at ambient temperature by magnetron co-sputtering of graphite and silicon circular targets (99.999% purity, 50 mm in diameter) in DCMS (graphite) and HiPIMS (silicon) modes. The angle between the symmetry axis of each magnetron and the normal to the surface of the deposition substrate was set to 22.5 degrees. Prior to the deposition process, the chamber of the sputtering system was evacuated to a base pressure lower than 10^−4^ Pa using a system consisting of a turbo-molecular pump and a dry scroll pump. Argon (99.999% purity) was utilized as a working gas, and it was supplied into the deposition chamber at a constant flow rate of 10 sccm.

Throughout the process, the gas pressure was varied from 0.5 to 2.0 Pa by controlling the valve between the chamber and the turbomolecular pump. The Si substrate was placed 100 mm away from the targets on the symmetry axis of the whole deposition system. No heating source was connected to the substrate holder during the sputtering process. The chemical composition and deposition rates of the films were calculated based on the calibrations performed for each sputtered target using a quartz crystal microbalance (QCM). By properly adjusting the average power applied on each target, the precise control of the films’ composition was obtained, independent of the processing gas pressure. The films’ thickness was controlled thanks to in situ monitoring of the deposition rate by the QCM.

The graphite sputtering process was performed in DCMS mode under a constant discharge power of 150 W. The silicon target was sputtered in pre-ionized HiPIMS mode under a constant power of 80 W using short voltage pulses with an amplitude of −950 V and a duration of 10 µs. An extensive description of the pre-ionized HiPIMS power supply and pre-ionized pulsed mode can be found in one of our previous works [21]. It should be mentioned that by varying the working gas pressure from 0.5 to 2.0 Pa, the peak current on the Si target remains constant at a value of 30 A. For each value of the process pressure, the pulsing frequency was correspondingly varied to preserve the stoichiometry of the deposited SiC films. In addition, to ensure the uniformity of the thickness and stoichiometry, the substrate holder was rotated during the deposition process around its symmetry axis, with a rotation speed of 10 rpm. Depending on the background processing gas pressure (p), the deposition rate (*S*) values ranged from 5.6 nm/min (p = 0.5 Pa) to 1.5 nm/min (p = 2 Pa) (see Table 1). The deposition time was conveniently adjusted to obtain SiC films with a thickness of about 750 nm.

### 2.2. Analytical Techniques

Atomic force microscopy (AFM) scans were performed with a NT-MDT Solver Pro system (Moscow, Russia) to obtain the morphology and roughness (R_RMS_ parameter) of the deposited SiC films. The surface was scanned in non-contact mode, ex situ, at ambient temperature using the same cantilever (NSC21 from Mikromasch, Tallinn, Estonia) and laser position to allow the qualitative comparison across the investigated samples. For statistical relevance, AFM scans were performed over multiple random 3×3 μm^2^ areas. Microscope controlling, image acquisition and data processing were performed using Nova software (version 1.0.26.1443) from NT-MDT.

The chemical composition and microstructure of the obtained SiC thin films were analyzed with a field-emission scanning electron microscope (NEON 40 EsB/Carl Zeiss AG, Oberkochen, Germany) equipped with an energy dispersive spectroscopy (EDS) detector (X-max from Oxford Instruments, Oxford, UK). Besides the detector, the analysis system includes a dedicated computer which acquires and processes the data by means of a dedicated software (INCA Energy Software 350, version 4.15) developed by Oxford Instruments. All the results (elemental analysis, EDS mapping, spectra, etc.) are generated through the INCA Energy software.

To investigate the chemical bonding configuration of the deposited SiC thin films, micro-Raman measurements were performed using a commercial confocal microscope (Renishaw inVia, Wotton-under-Edge, Gloucestershire, United Kingdom) equipped with a He-Ne laser (excitation wavelength—632.8 nm, beam power—17 mW) and a CCD detector coupled to a Leica DM 2500M microscope (Wetzlar, Germany). All the measurements were carried out under ambient laboratory conditions (ambient temperature and atmospheric pressure) in backscattering geometry, with the light being focused and collected through a 50× objective with a numerical aperture of 0.75. Spectral data processing was performed with the WiRE 3.2 software (Renishaw, UK).

The structural properties of the SiC thin films were analyzed by X-ray diffraction (XRD) using a Shimadzu LabX XRD-6000 Diffractometer (Kyoto, Japan) with Cu K_α_ radiation (λ = 1.54059 Å) in Bragg–Brentano configuration. The diffractograms were recorded in the 2θ range of 10–80 degrees with a scan speed of 0.6 deg/min.

The mechanical and tribological properties (Young’s modulus, E, hardness, H, critical adhesion loads, LC, and coefficient of friction) of SiC films deposited on Si substrates were investigated by instrumented indentation and micro-scratch tests in ambient atmosphere using a nanoindentation tester (NHT^2^, equipped with a three-sided diamond pyramidal Berkovich indenter tip, tip radius of about 100 nm) and a micro-scratch tester (MST, equipped with a diamond Rockwell-type indenter, tip radius of about 100 μm) from CSM Instruments/Anton Paar (Peseux, Switzerland). The nanoindentation data was processed using the Oliver&Pharr model [22]. To minimize the substrate’s influence on the indentation data, the maximum load was set in such a way as to limit the indentation depth to no more than 100 nm (approximately 15% of the films’ thickness). In addition, for statistical relevance, the load-controlled indentations were performed over multiple random areas, and the average values of hardness and Young’s modulus were calculated from at least 30 load-displacement curves. During microscratch tests, the load was progressively applied on a length of 3 mm, from 0.03 N to 10.00 N, with a loading rate of 5 N/min. The critical load values were determined by microscopic analysis of the residual scratch tracks. The critical load values given for each sample are the average of at least five scratch tracks. These critical adhesion loads are defined as follows: the first critical load (LC_1_)—the load necessary for the emergence of the first cracks in the film; the second critical load (LC_2_)—the load corresponding to the first delamination of the film; and the third critical load (LC_3_)—the load necessary for the delamination of more than 50% of the film from the scratch track. The wear behavior was assessed on the same micro-scratch tester (MST, Anton Paar/CSM Instruments, Peseux, Switzerland) using the following parameters: Si_3_N_4_ 5 mm balls as counter friction partner; dry conditions; applied load—1N; linear motion; wear track length 5 mm; stop condition—33 passes. The result of interest was the variation of the dynamic friction coefficient as a function of the pass number.

## 3. Results and Discussion

### 3.1. Surface Morphology

**Figure 1:**
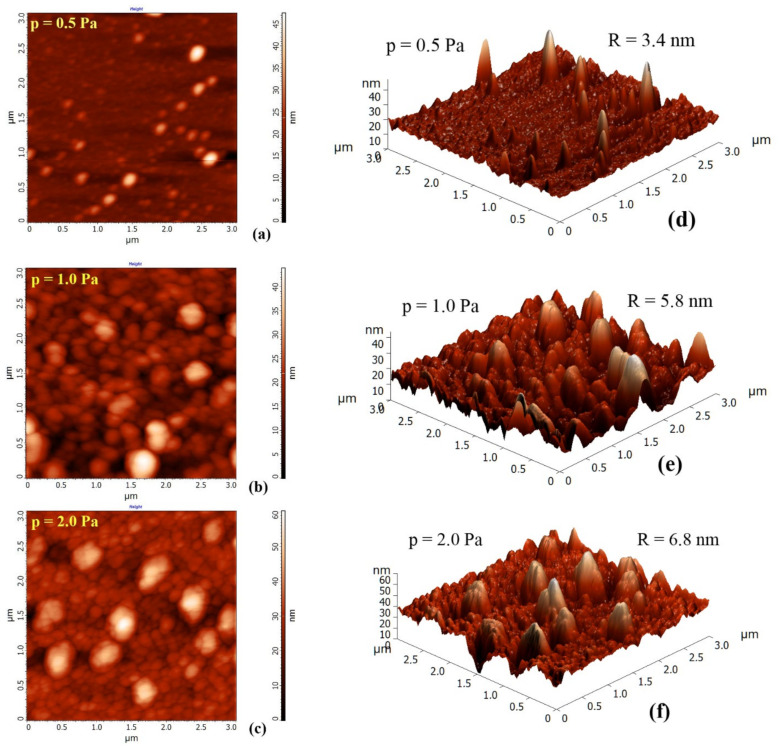
AFM images in 2D and 3D showing the surface morphology of SiC films deposited under 3 different process gas pressures: 0.5, 1.0, and 2.0 Pa, respectively: 2D images (**a**) p = 0.5 Pa, (**b**) p = 1.0 Pa, (**c**) p = 2.0 Pa, and 3D images (**d**) p = 0.5 Pa, (**e**) p = 1.0 Pa, (**f**) p = 2.0 Pa.

Figure 1a–f present 2D (Figure 1a–c) and 3D (Figure 1d–f) AFM images of 3 selected SiC films showing the influence of the argon pressure on the morphology of their surfaces. These images reveal that the deposited SiC films grow in grain/columnar mode and have compact structures. As can be seen, uneven surface grains are observed, and their size and, consequently, the root mean square roughness (R_RMS_), gradually increases as the gas pressure increases. The gas scattering effect explains well this behavior: the number of gas-phase collisions in the path from the targets to the substrate increases with the gas pressure, leading to a decrease in the mean free path. This scattering effect leads to a decrease in the kinetic energy of the sputtered particles, increasing their angular distribution. This effect determines the reduction of the deposition rate. The increased angular distribution enhances the shadowing effect, while the reduced kinetic energy lowers the mobility of the ad-atoms on the surface of the growing film [23]. According to the relationship between the process pressure and kinetic energy of the sputtered particles, under low gas pressure, the sputtering process is mainly ballistic. Consequently, the sputtered particles can reach the substrate with few or no collisions in the gas phase, and due to sufficient energy diffusion and migration, they can fill the voids in the film’s volume, leading to smooth thin films.

### 3.2. Chemical Composition

The chemical composition of the deposited SiC thin films was accurately measured by EDX. Table 1 (previously presented) summarizes the atomic percentages of silicon and carbon measured on each SiC thin film deposited under different working gas pressure conditions. EDX results clearly show that all the deposited SiC thin films are near stoichiometric.

### 3.3. Structural Properties

#### 3.3.1. Raman Spectroscopy

Raman scattering was used to investigate the influence of the working gas pressure on the chemical bonding configuration of silicon carbide thin films. Raman spectroscopy is a powerful non-destructive characterization tool for silicon carbide due to its capability to identify atomic polar bonds, such as Si–Si, C–C, and Si–C, as well as Si and C clusters embedded in the SiC thin films. The Raman spectra of the as-deposited SiC samples were acquired within the 400–1800 cm^−1^ spectral range.

**Figure 2:**
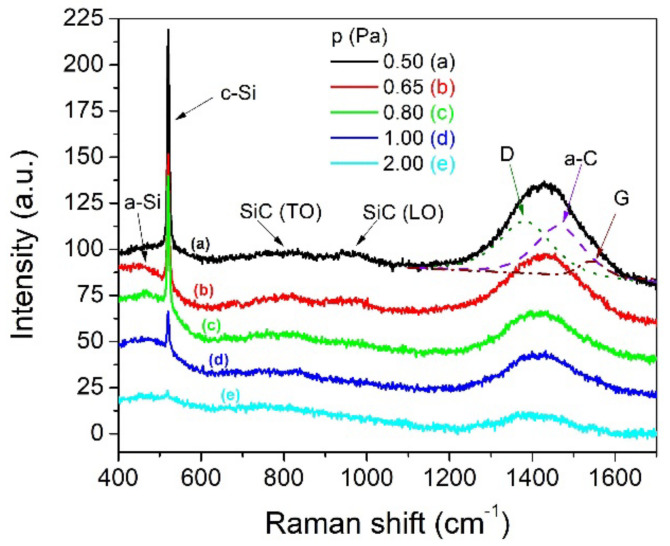
Raman spectra of SiC thin films deposited on Si substrates using DCMS/HiPIMS co-sputtering technique under different process pressures (a) p = 0.50 Pa, (b) p = 0.65 Pa, (c) p = 0.80 Pa, (d) p = 1.00 Pa, (e) p = 2.00 Pa.

As shown in Figure 2, the first band, positioned around 480 cm^−1^, corresponds to the Si–Si transverse optical (TO) mode from amorphous Si. A TO mode of nanocrystalline Si (c-Si) sharp peak appears at 520 cm^−1^. As the processing gas pressure decreases, the intensity of the c-Si peak increases. The enhanced intensity at lower gas pressure may be due to the increased energy and mobility of ad-atoms (see discussion from Section 3.1). Lattemann et al. [20] showed that the intensity of the c-Si peak steeply increases as the substrate bias increases due to the enhanced energy of the deposited ions. The broad and weak band, which peaked at approximately 790 cm^−1^, is typical for the TO mode of nanocrystalline SiC, while the band centered at 968 cm^−1^ belongs to the longitudinal optic (LO) mode of the A1 symmetry of SiC [24]. Nakashima et al. [25] reported a similar feature, and they attributed it to cubic (TO mode) and hexagonal (LO mode) SiC polytypes, respectively.

The broad band situated between 1200 and 1600 cm^−1^ is related to C–C vibrational modes. The Gaussian fitting of this broad band reveals 3 peaks: 2 typical peaks of amorphous carbon materials (D peak at 1380 cm^−1^ and G peak at 1550 cm^−1^) and an a-C peak centered at 1450 cm^−1^ [26]. The G peak is directly related to the bond stretching of sp^2^ sites in both graphitic ring and olefinic chain structures, a common feature of all disordered carbons (sp^3^ hybridization), while the D peak is an indication that the *sp*^2^ sites are organized only into graphitic rings. DLC (diamond-like carbon) with higher *sp*^3^ content tends to have more chain structures, which are linked to higher mass density and hardness. As compared to the D peak, the intensity of the G peak is much lower because the *sp*^3^ coordinated amorphous carbon is hard to directly observe in visible Raman [27]. The band detected at 1450 cm^−1^ corresponds to a precursor phase of the tetrahedrally bonded carbon, and it is a sign of partial crystallization of carbon clusters [28].

Even if the SiC coatings are near stoichiometric, the relative intensity of the Si-C bands is very low, and the carbon bands dominate the Raman spectra. The much higher scattering cross-section and absorption coefficient of the C–C bonds as compared to the Si–C bonds seems to be the main reason for this behavior [29]. By decreasing the gas pressure, the intensities of C, SiC, and c-Si bands increase, highlighting the enhancement of the density of bonding states and the improvement of short-range order. The Raman spectra of coatings deposited at 0.5, 0.65 and 0.8 Pa look the same as those reported by Craciun et al. [30] for near stoichiometric and nanocrystalline SiC layers deposited by pulsed laser deposition at a nominal substrate temperature of 1000 °C.

#### 3.3.2. X-ray Diffractometry

**Figure 3:**
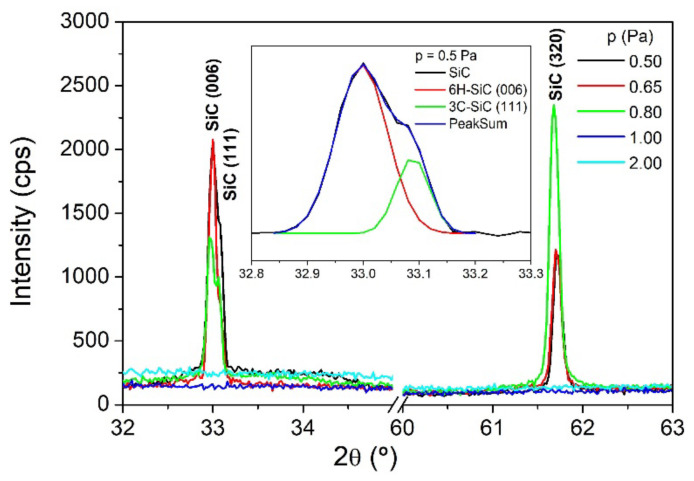
XRD patterns of SiC thin films deposited under different gas pressures. The inserted graph displays the deconvolution of the SiC peak (2θ = 33°) observed for the sample deposited at 0.5 Pa.

The structure of the magnetron-sputtered silicon carbide thin films was investigated by carrying out X-ray diffraction measurements. Figure 3 displays XRD patterns for the as-deposited SiC thin films. Analyzing these diffraction patterns, it can be seen that the samples deposited with 1 and 2 Pa argon pressure are amorphous, while the samples deposited with 0.50, 0.65, and 0.80 Pa argon pressure are composed of a mixture of hexagonal, cubic and rhombohedral SiC phases.

A nanocrystalline structure (but with different phases) was recently reported by Galvão et al. [19] for SiC films deposited by HiPIMS at ambient temperature. The XRD results reported in this paper are more consistent with those reported by Craciun [30] and Keffous [31]. The peak positioned at 33.0° corresponds to the hexagonal phase 6H-SiC (006) and cubic phase 3C-SiC (111) [30], while the peak from 61.7° is assigned to the rhombohedral SiC (320) phase [31]. As the gas pressure increases, the films’ structure changes from a hexagonal-preferential orientation to a rhombohedral-preferential orientation.

The XRD results are well correlated with Raman data, which show a gradual decrease of SiC bands, corresponding to hexagonal and cubic phases, by increasing the Ar pressure in the deposition chamber. The intense energetic ion bombardment during the magnetron sputtering of the Si target can contribute to a nano-size refinement of the crystallites by means of re-nucleation, and it can also explain the lack of other significant 6H-SiC peaks. XRD data highlight the fact that ion bombardment (controlled by the working gas pressure) can modify the growth mechanism of the SiC thin films, and it can also strongly influence their crystalline structure and degree of crystallinity. By decreasing the gas pressure, due to a larger mean free path, the sputtered particles preserve their intrinsic energy, which in turn results in improved ad-atom mobility and enhanced atomic short-range order.

### 3.4. Mechanical Properties

The process pressure has a significant influence on the mechanical and tribological performance of the deposited SiC thin films. Comparative data concerning the determined values of the mechanical and tribological parameters (hardness, Young’s modulus, critical adhesion loads) of SiC films deposited on Si substrates at ambient temperature with different values of the working gas pressure are listed in Table 2.

**Figure 4:**
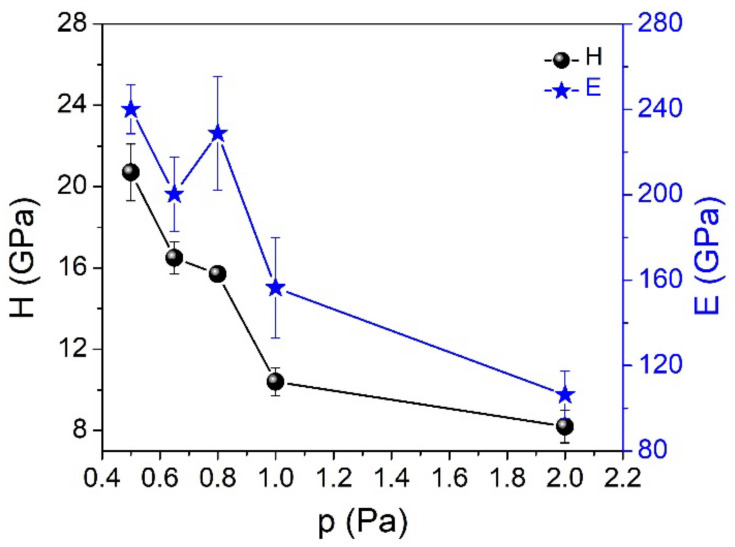
The variation of hardness and Young’s modulus of SiC thin films as a function of the process pressure.

When the argon pressure is increased from 0.5 to 2.0 Pa, the hardness and Young’s modulus gradually decrease from 20.7 to 8.2 GPa (H) and from 240 to 106 GPa (E), respectively (see Figure 4). This behavior can be associated with the structural evolution of the films, their atomic-range order and the Si-C bond density. This change is also influenced by the creation of numerous growth defects (micro-voids, dangling bonds and structural disorders, such as Si or C clusters embedded in the SiC films). In the case of completely amorphous SiC films formed under high gas pressure (2 Pa), the low hardness value may be due to their low densification degree. The increase of the gas pressure results in reduced kinetic energy and mobility of the ad-atoms on the surface of the growing film and, consequently, in a less dense structure and a lower short-range order.

Both hardness and Young’s modulus can be significantly enhanced by increasing the deposition temperature, which leads to an improvement in the short-range order and partial crystallization of the film [32]. However, an analysis of the influence of this deposition parameter was beyond the scope of the present work. On the other hand, the hardness and Young’s modulus of the SiC films are strongly related to their Si-C bond density. El Khakani [33] showed that H and E values are strongly related to the Si-C bond density in SiC films. An increase in the Si-C bond density results in a strengthening of atomic bonds and, consequently, in an improvement in their mechanical and tribological performance.

**Figure 5:**
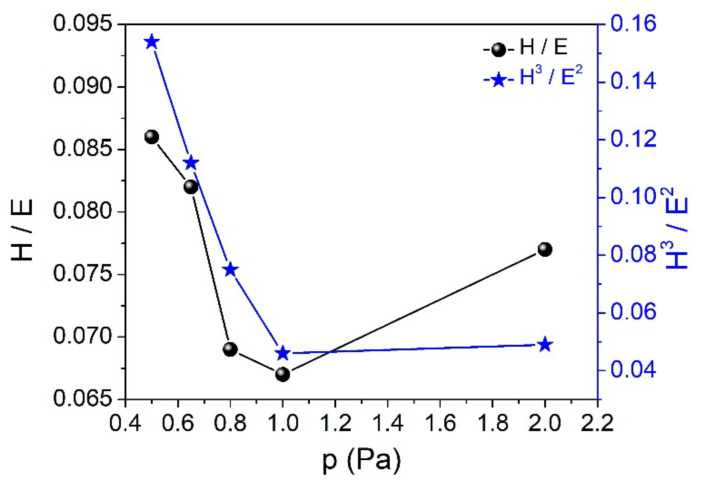
The variation of H/E and H^3^/E^2^ ratios of SiC thin films as a function of the process pressure.

The hardness and Young’s modulus have a significant influence on the wear behavior, for instance. The resistance to elastic deformation (elastic strain to failure), represented by the H/E ratio, has been used to predict the wear resistance of a material [34,35]. With the exception of the case of the sample produced under 2.0 Pa argon pressure, the H/E ratio increases when the gas pressure decreases (Figure 5), especially when the polycrystalline SiC phase starts to be formed in the amorphous matrix (see XRD and Raman results). In other words, the hardness enhancement is probably due not only to the Young’s modulus enhancement, but also due to the formation of specific nanostructures (crystalline phases) which prevent the plastic deformation of the coating. The nano-size refinement of the crystallites by re-nucleation during the deposition process prevents pronounced growth of the grains, as can be clearly seen from the AFM images.

The H^3^/E^2^ ratio helps to estimate what happens in terms of dissipation of energy when the film suffers plastic deformation under a certain load [36]. The obtained results match the results of the H/E ratio. The films with higher resistance to elastic deformation, produced under p = 2.0 Pa argon pressure, are those that dissipate more energy when plastic deformation occurs. The H/E^2^ ratio gives information about the resistance of the material to permanent damage [37]. The variation of this ratio as a function of the deposition pressure (not shown here) is the opposite of the other two ratios. Those films exhibiting lower resistance to elastic deformation are those that may have higher resistance to permanent damage, according to these ratios.

**Figure 6:**
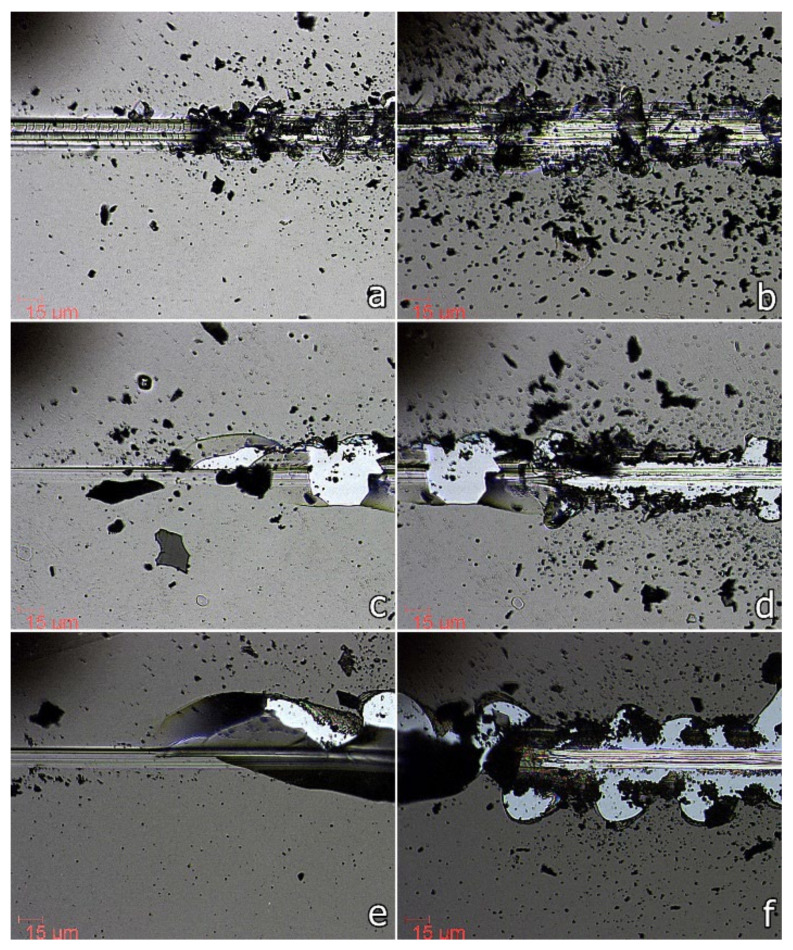
Optical micrographs of scratch tracks showing different stages of material failure (LC2—adhesive failure—left, and LC_3_—delamination failure—right) for SiC thin films deposited by DCMS/HiPIMS co-sputtering technique on silicon substrates under different working gas pressures: LC2—(**a**) p = 0.5 Pa, (**c**) p = 1.0 Pa, (**e**) p = 2.0 Pa; LC3—(**b**) p = 0.5 Pa, (**d**) p = 1.0 Pa, (**f**) p = 2.0 Pa.

Generally, the produced SiC films exhibited good cohesion, since no cohesive failures (cracks in the coatings) were detected at the beginning of the scratch tests prior to adhesive failure. Figure 6 presents optical micrographs of different stages (LC_2_—left micrograph column, and LC_3_—right micrograph column) showing the dynamics of the SiC film—Si substrate system failure and the impact of the working gas pressure on the coating/substrate interfacial behavior.

**Figure 7:**
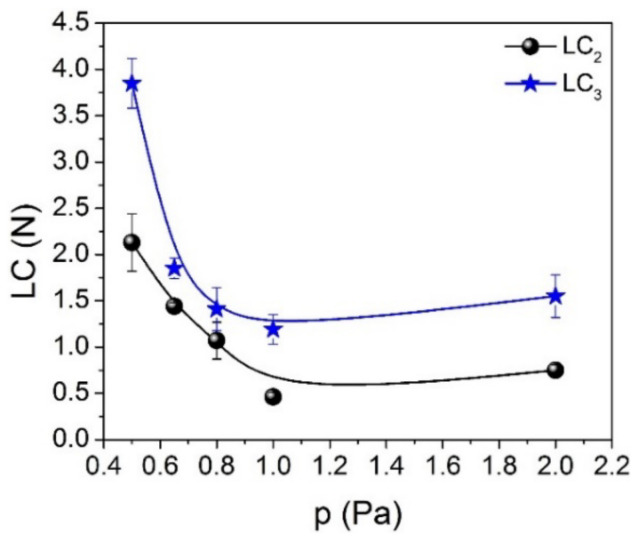
The variation of critical adhesion loads (LC_2_—adhesive failure, and LC_3_—delamination failure) as a function of the working pressure for SiC thin films deposited by the DCMS/HiPIMS co-sputtering technique on silicon substrates.

The variation of both critical adhesion loads, LC_2_ and LC_3_, when the working gas pressure was increased from 0.5 to 2.0 Pa, is in good agreement with the trend observed for H/E and H^3^/E^2^ ratios, especially for the 0.5–1.0 Pa region (Figure 5 and Figure 7). Increasing the gas pressure from 0.5 to 2.0 Pa, the first adhesive failure (LC_2_) varies from 2.13 to 0.75 N, reaching its minimum value (0.46 N) at 1.0 Pa Ar pressure (see Table 2 and Figure 7). The slight increase of LC_2_ value in the 1.0–2.0 Pa region could be due to an increase in the resistance to permanent damage which, as we mentioned before, was found to have an opposite trend to the H/E and H^3^/E^2^ ratios. In general, a better wear behavior is attributed not only to hardness itself, but rather to the H/E ratio [34]. In this work, when the working gas pressure is increased from 0.5 to 2.0 Pa, the H/E value varies from 0.086 to 0.077, showing a minimum of 0.066 for the samples deposited under 1.0 Pa Ar pressure.

The internal stress caused by the large lattice mismatch (20%) between the SiC coatings and Si substrates and different thermal expansion coefficient (8%) may be a potential reason for the relatively low LC values found for the deposited SiC films. It should be mentioned that the coatings presented in this work were obtained without any substrate treatments (interlayer, etching, ion implantation, biasing, heating, annealing). Usually, the adhesion of a film on a substrate may be improved if one of these treatments is performed. The use of interlayers can improve the film’s adhesion due to reduced thermal stress between SiC and Si materials [38].

**Figure 8:**
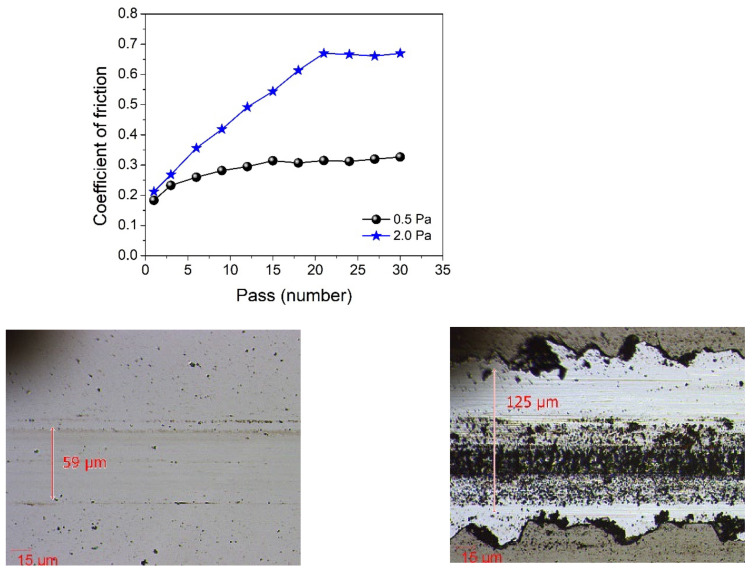
The variation of the friction coefficient as a function of the pass number for the lowest and highest-pressure SiC samples and images of characteristic wear zones (left image corresponds to the sample deposited under gas pressure of 0.5 Pa, while the right one corresponds to 2.0 Pa).

Figure 8 presents the variation of the friction coefficient as a function of the number of passes for the lowest and highest-pressure samples, namely 0.5 Pa and 2.0 Pa and images representing characteristic wear zones captured after the tests. One can observe that, even if the starting friction coefficient, for the first few passes, has relatively similar values, once the silicon nitride ball passes multiple times on the same wear track, the friction coefficient rises significantly for the 2.0 Pa sample.

This phenomenon is most likely caused by one or a combination of several factors: (i) significant differences in the H/E ratio; (ii) much lower adhesion critical loads for the 2.0 Pa sample (delamination of the coating and presence of wear debris on the wear track); (iii) differences in surface roughness and the film’s microstructure.

**Figure 9:**
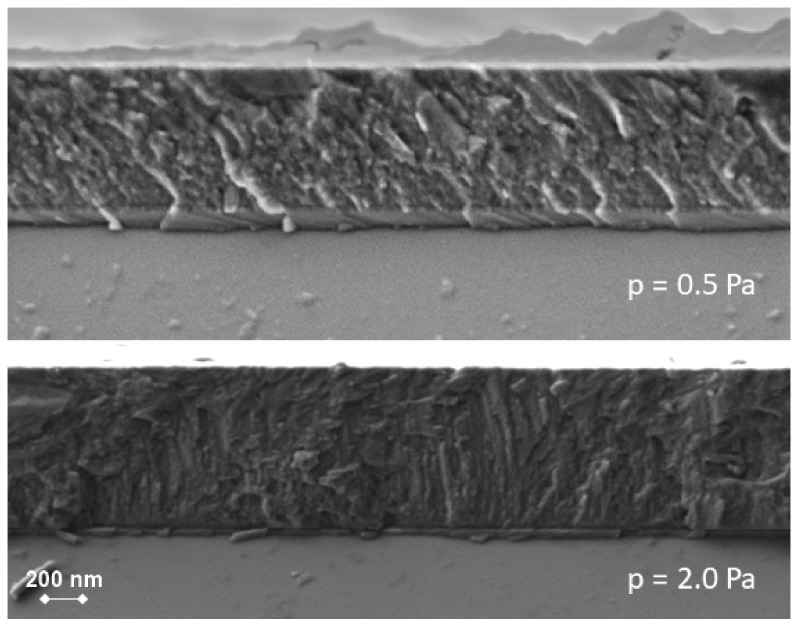
Cross-sectional SEM images of SiC thin films deposited at 0.5 and 2.0 Pa, respectively.

According to cross-sectional SEM images (Figure 9), a denser and more homogeneous film tends to be formed under lower processing gas pressure. The increase of the process pressure leads to a columnar and more porous structure in the film, which, in turn, results in a decrease of the abrasive wear resistance.

## 4. Conclusions

This study showed that both structural and mechanical properties of SiC thin films can be significantly influenced and tailored during DCMS/HiPIMS co-sputtering deposition if the working gas (argon) pressure is varied over a range of 0.5–2.0 Pa. It has also been shown that the DCMS/HiPIMS co-sputtering technique allows the deposition of near stoichiometric and nanocrystalline SiC thin films without heating or biasing the substrate. The obtained results indicate a strong correlation between films’ microstructure and mechanical properties. Decreasing the process pressure leads to low surface roughness, dense structure and an enhancement in the short-range order and bonding states of SiC thin films, resulting in superior mechanical and tribological performance.

## Figures and Tables

**Table 1:** Values of deposition rate (S), roughness (R_RMS_), and atomic concentration of SiC films deposited on Si substrates using different process pressures.

Sample	p (Pa)	S (nm/min)	R_RMS_^a^ (nm)	Si ^b^ (at.%)	C ^b^ (at.%)	Si/C
SiC_1	0.50	5.6	3.4	49 ± 1	51 ± 1	0.96
SiC_2	0.65	4.6	4.4	49 ± 1	51 ± 1	0.96
SiC_3	0.80	3.4	5.0	49 ± 1	51 ± 1	0.96
SiC_4	1.00	3.0	5.8	49 ± 1	51 ± 1	0.96
SiC_5	2.00	1.5	6.8	52 ± 2	48 ± 2	1.08

^a^ Referred to in Section 3.1, ^b^ Referred to in Section 3.2.

**Table 2:** Values for hardness (H), Young’s modulus (E), H/E and H^3^/E^2^ ratios, and critical adhesion loads (LC) for SiC films deposited on silicon substrates using different process pressures.

Sample	p (Pa)	H (GPa)	E (GPa)	H/E	H^3^/E^2^	LC_2_ (N)	LC_3_ (N)
SiC_1	0.50	20.7 ± 1.4	240 ± 11	0.086 ± 0.010	0.154 ± 0.045	2.13 ± 0.31	3.85 ± 0.27
SiC_2	0.65	16.5 ± 0.8	200 ± 17	0.082 ± 0.011	0.112 ± 0.035	1.44 ± 0.04	1.85 ± 0.11
SiC_3	0.80	15.7 ± 0.3	229 ± 27	0.069 ± 0.009	0.075 ± 0.022	1.07 ± 0.20	1.41 ± 0.23
SiC_4	1.00	10.4 ± 0.7	156 ± 24	0.066 ± 0.015	0.046 ± 0.023	0.46 ± 0.05	1.19 ± 0.16
SiC_5	2.00	8.2 ± 0.8	106 ± 11	0.077 ± 0.016	0.049 ± 0.025	0.75 ± 0.06	1.55 ± 0.23

## Data Availability

The data is available on reasonable request from the corresponding author.
